# Unraveling gene content variation across eukaryotic giant viruses based on network analyses and host associations

**DOI:** 10.1093/ve/veab081

**Published:** 2021-09-16

**Authors:** Tsu-Wang Sun, Chuan Ku

**Affiliations:** Institute of Plant and Microbial Biology, Academia Sinica, Taipei 11529, Taiwan; Genome and Systems Biology Degree Program, Academia Sinica and National Taiwan University, Taipei 10617, Taiwan; Institute of Plant and Microbial Biology, Academia Sinica, Taipei 11529, Taiwan; Genome and Systems Biology Degree Program, Academia Sinica and National Taiwan University, Taipei 10617, Taiwan

**Keywords:** animal, genome evolution, phylogenomics, protein families, protist, virus–host interaction

## Abstract

The nucleocytoplasmic large DNA viruses (NCLDVs, phylum Nucleocytoviricota) infect vertebrates, invertebrates, algae, amoebae, and other unicellular organisms across supergroups of eukaryotes and in various ecosystems. The expanding collection of their genome sequences has revolutionized our view of virus genome size and coding capacity. Phylogenetic trees based on a few core genes are commonly used as a model to understand their evolution. However, the tree topology can differ between analyses, and the vast majority of encoded genes might not share a common evolutionary history. To explore the whole-genome variation and evolution of NCLDVs, we dissected their gene contents using clustering, network, and comparative analyses. Our updated core-gene tree served as a framework to classify NCLDVs into families and intrafamilial lineages, but networks of individual genomes and family pangenomes showed patterns of gene sharing that contradict with the tree topology, in particular at higher taxonomic levels. Clustering of NCLDV genomes revealed variable granularity and degrees of gene sharing within each family, which cannot be inferred from the tree. At the level of NCLDV families, a correlation exists between gene content variation, but not core-gene sequence divergence, and host supergroup diversity. In addition, there is significantly higher gene sharing between divergent viruses that infect similar host types. The identified shared genes would be a useful resource for further functional analyses of NCLDV–host interactions. Overall this study provides a comprehensive view of gene repertoire variation in NCLDVs at different taxonomic levels, as well as a novel approach to studying the extremely diverse giant virus genomes.

## 1. Introduction

The nucleocytoplasmic large DNA viruses (NCLDVs) are double-stranded DNA viruses widely found in eukaryotes and constitute the recently established virus phylum Nucleocytoviricota (Koonin, Dolja, and Krupovic et al., 2020). Commonly known as giant viruses (Van Etten and Meints 1999; Fischer 2016; Wilhelm et al., 2016), they are characterized by the largest virion and genome size in the virus world, some even with Mb-sized genomes (Raoult et al., 2004; Philippe et al., 2013; Andreani, Khalil, and Baptiste et al., 2018). NCLDVs are associated with various major lineages of eukaryotes (Sun, Yang, and Kao et al., 2020; Meng, Endo, and Blanc-Mathieu et al., 2021), often as prominent components of the eukaryotic virosphere in diverse environments (Carradec, Pelletier, and Da Silva et al., 2018; Schulz, Alteio, and Goudeau et al., 2018). They are key regulators of host population dynamics, with important ecological, agricultural, and health impacts, and recently they have been shown to shape host chromosomes through endogenization of their DNA (Gallot-Lavallée and Blanc 2017; Moniruzzaman, Weinheimer, and Martinez-Gutierrez et al., 2020b; Nelson, Hazzouri, and Lauersen et al., 2021). However, the evolution of NCLDV genomes still remains poorly understood, in particular regarding the relationships among divergent NCLDV families and their gene content evolution, which could have important implications for the debate over their origin(s) and evolutionary relationships with cellular organisms (Yutin, Wolf, and Koonin 2014; Moreira and López-García 2015; Ku and Sun 2020).

Evolutionary relationships among NCLDVs have been most commonly represented by phylogenetic trees of individual protein-coding genes or a combined set of genes that are widely shared across NCLDVs (i.e. core genes). This approach has been instrumental in characterizing new NCLDVs and defining a species or genus comprised by closely related strains that generally infect a particular host. It has also been used to delineate families of NCLDVs and to resolve interfamilial relationships (Koonin and Yutin 2018; Guglielmini, Woo, and Krupovic et al., 2019), where there is still no general agreement between studies. This underlines the limitations of the core-gene phylogenetics approach, especially given the paucity or lack of universally shared and strictly vertically inherited genes across divergent viruses as a result of viral evolution (Yutin and Koonin 2012; Claverie 2020). In addition, even if a core-gene tree can accurately depict the relationships among NCLDVs and their families, the tree topology cannot directly translate into the evolutionary history of all the non-core genes that constitute the vast majority of the NCLDV coding capacity.

An alternative approach is to take into account all the coding sequences in whole genomes. Mapping of gene presence–absence patterns onto core-gene trees has revealed extensive gene gains and losses across NCLDV lineages and multiple origins of NCLDV genome gigantism (Yutin, Wolf, and Koonin 2014; Koonin and Yutin 2018). These gene content variations can result from accordion-like duplications and losses of existing genes (e.g. a poxvirus protein involved in counteracting host defense (Elde, Child, and Eickbush et al., 2012)) and acquisitions of genes with various functions through lateral transfers from hosts or host-associated microbes (Filée, Pouget, and Chandler 2008; Sun, Yang, and Kao et al., 2020). Genome-wide gene contents can also be used to infer phylogenetic trees of NCLDV genomes, which are largely congruent with core-gene trees in familial delineation, but they tend to differ in interfamilial branching patterns (Yutin, Wolf, and Koonin 2014; Legendre, Fabre, and Poirot et al., 2018; Needham, Poirier, and Hehenberger et al., 2019; Yoshikawa, Blanc-Mathieu, and Song et al., 2019). Despite its simplicity, tree-like representation might not be the best way to resolve the complex evolutionary relationships among NCLDV genomes. Another option is network-based models, which are especially useful for the study of microbial genomes that undergo frequent reticulate evolutionary processes (Dagan and Martin 2009; Corel, Lopez, and Méheust et al., 2016). Network analyses have been successfully applied to resolve the connections among double-stranded DNA viruses and among metagenomically assembled NCLDV genomes (Iranzo, Krupovic, and Koonin 2016; Schulz, Alteio, and Goudeau et al., 2018; Moniruzzaman, Martinez-Gutierrez, and Weinheimer et al., 2020a). However, these methods have not been extensively explored for elucidating the relationships among NCLDV lineages and families.

The commonly delineated NCLDV families show enormous variation not only in their gene contents, but also in their host diversity. For example, the only known host of *Marseilleviridae* is the amoebozoan genus *Acanthamoeba* (Doutre, Philippe, and Abergel et al., 2014), whereas *Mimiviridae* hosts encompass most supergroups (highest-level taxa) of eukaryotes (Sun, Yang, and Kao et al., 2020; Meng, Endo, and Blanc-Mathieu et al., 2021). Host associations are a crucial factor in genome evolution of NCLDVs, given that host biology shapes viral replication and adaptation and determines the ecological environment and potential sources of lateral gene transfers. It has also been suggested that heterotrophic or phototrophic lifestyles of hosts can influence gene contents of giant viruses (Needham, Poirier, and Hehenberger et al., 2019). These indicate a need to more comprehensively examine how host diversity correlates with genomic variation and whether viruses infecting eukaryotes with similar ecological traits or more phylogenetically related tend to share more genes.

The number of NCLDV genomes sequenced grows rapidly each year. Here we took advantage of available complete and near-complete genome sequences of NCLDVs, especially those with known hosts, and applied gene clustering, phylogenetics, network analyses, and comparative methods to better illuminate their genomic variation and evolution. The focal point of this study is well recognized—yet poorly understood—taxonomic families of NCLDVs, with particular emphasis on their gene contents, host associations, and interfamilial relationships. Through the comprehensive approach presented in this study, we move beyond core-gene phylogenies and provide novel insights into virus–host interactions and their impacts on NCLDV evolution.

## 2. Methods

### 2.1 Genomic data

We collected NCLDV sequence data listed in the National Center for Biotechnology Information (NCBI) Virus (https://www.ncbi.nlm.nih.gov/labs/virus/) database (as of August 2019), and other published sequences not listed there were retrieved from NCBI GenBank (Benson, Karsch-Mizrachi, and Clark et al., 2012). The finalized dataset includes protein-coding sequences from 196 viruses with known hosts and 11 metagenomically assembled genomes (MAGs) across NCLDV families that have been proposed: *Ascoviridae, Asfarviridae, Iridoviridae, Marseilleviridae, Medusaviridae, Mimiviridae, Molliviridae, Pandoraviridae, Phycodnaviridae, Pithoviridae*, and *Poxviridae*. Protein sequences, annotations, and metadata of the viruses were collected from NCBI GenBank and Virus databases, as well as Virus-Host DB (https://www.genome.jp/virushostdb/) and relevant publications (Supplementary Table S1). The genome size and number of protein-coding genes were calculated for each genome and listed in Supplementary Table S1.

### 2.2 Ortholog clustering

The protein sequences were extracted from all 207 genomes, with each renamed as ‘VirusID|protein_accession’ (Supplementary Dataset S1), where the VirusID is unique for each viral genome as listed in Supplementary Table S1. Sequences shorter than 10 residues were removed from the dataset. An all-against-all search was conducted using BLAST v2.6.0 (Altschul et al., 1997), with an expect value below 10^−5^, to quantify the protein similarities, which were used to cluster the sequences by OrthoMCL v1.3 (Li, Stoeckert, and Roos 2003) into orthologous gene clusters (hereafter orthogroups) with an inflation of 1.1.

### 2.3 Core-gene phylogeny

Protein sequences were annotated through similarity searches using DIAMOND v0.9.24.125 (Buchfink, Xie, and Huson 2015) with an expect value below 10^−5^ against the Nucleo-Cytoplasmic Virus Orthologous Groups (NCVOG) database (Yutin, Wolf, and Koonin 2014; Schulz, Alteio, and Goudeau et al., 2018). Based on the NCVOG annotations, we identified gene orthogroups corresponding to the five core proteins used for phylogenetic analyses in a previous study (Schulz, Yutin, and Ivanova et al., 2017). Protein sequences from these orthogroups were aligned using MAFFT v. 7.310 (Katoh and Standley 2013), where the longest sequence was used to represent genomes with more than one homolog in an orthogroup. A maximum-likelihood phylogenetic tree of the concatenated alignments was constructed using IQ-TREE v. 2.1.3 (Minh, Schmidt, and Chernomor et al., 2020) with the Q.pfam+F+R9 model selected by ModelFinder (Kalyaanamoorthy, Minh, and Wong et al., 2017) and with ultrafast bootstrap (Hoang, Chernomor, and Von Haeseler et al., 2018) branch support values estimated using 1,000 replicates.

### 2.4 Network analyses of gene sharing

We constructed networks of gene sharing among viral taxa—either individual NLCDVs or taxonomic families of NCLDVs—based on their presence/absence in each of the orthogroups. For families, all genes encoded by viruses in the same family were considered as one pangenome. We define the level of gene (orthogroup) sharing (*S*) between two taxa *i* and *j* as the number of orthogroups they share (*U*) normalized by the geometric mean of their respective total numbers of orthogroups shared with any taxon (*T*):


$$\begin{equation*}{S_{ij}} = {{{U_{ij}}} \over {\,\sqrt {{T_i} \times {T_j}} }}.\end{equation*}$$

To take into account the gene repertoire size of both taxa while avoiding the overinfluence of a much larger size than the other, their geometric mean, instead of arithmetic mean, was used as the normalization factor.

Cytoscape v3.8.2 (Shannon et al., 2003) was used to analyze and visualize gene sharing patterns among NCLDVs, with taxa specified as nodes and the level of gene sharing as edge attributes. Individual NCLDV genomes were clustered using the Markov Clustering Algorithm (MCL) (Enright, Van Dongen, and Ouzounis 2002) with a granularity index of 1.5. The gene sharing patterns within clusters of individual NCLDVs or among NCLDV families were visualized using the Prefuse Force Directed Layout (Heer, Card, and Landay 2005).

### 2.5 Genomic variation and host diversity of NCLDV families

To have an overall understanding of genomic variation and host diversity at the family level, we explored three measures of intrafamilial genomic variation and plotted them against a phylogenetic diversity index of hosts. Based on the core-gene phylogeny, each family was divided into distinct intrafamilial lineages (Supplementary Table S1), with each lineage consisting of one to many most related genomes (e.g. those from the same genus). By grouping closely related genomes into lineages before quantifying intrafamilial genomic variation, we avoided the effects of oversampling closely related strains from the same lineage due to their biased sequence availability in the databases. For each lineage with two or more genomes, we obtained the average across its individual genomes before calculating the intrafamilial, between-lineage variation. These intrafamilial genomic variation measures include (1) the standard deviation of protein-coding sequence counts across lineages within a family; (2) the standard deviation of unclustered singleton sequence counts across lineages within a family; and (3) the average pairwise distance (branch length in substitutions per site) in the core-gene tree (Section 2.3) between lineages within a family. To quantify the phylogenetic diversity of hosts across lineages within a family, we considered the host distribution across the major lineages, or supergroups, of eukaryotes (Adl, Bass, and Lane et al., 2019), including Amoebozoa, Archaeplastida, Discoba, Haptista, Opisthokonta, and the grouping of Stramenopila, Alveolata, and Rhizaria (SAR) that have known hosts of NCLDVs (Sun, Yang, and Kao et al., 2020). Based on the Shannon index, the host diversity of a family (*D*) was calculated from the proportions of lineages (*p*) that infect a certain eukaryote supergroup (*j*) out of the six (*n*):


$$\begin{equation*}D = - \sum\limits_j^n {{p_j}\;\ln \;{p_j}} .\end{equation*}$$

### 2.6 Gene sharing between viruses with similar hosts

We investigated the relationships between host associations and gene contents by comparing the level of gene sharing between viruses with similar or dissimilar host types in terms of phylogenetic and eco-physiological attributes. Two pairs of virus families were chosen that have adequate numbers of viruses infecting similar types of host: *Iridoviridae*–*Poxviridae* (mainly infecting vertebrates and insects) and *Mimiviridae*–*Phycodnaviridae* (mainly infecting algae and amoebae). The level of gene sharing was calculated for pairs of viruses where each is from a different family in a pair of virus families, which gives the advantage of disentangling the effects of host associations from phylogenetic relatedness. For each of the four host types, the calculation was done for all pairs of viruses where both viruses from the two families infect this host type (similar hosts) or where one infects this host type and the other infects any other host types (dissimilar hosts). A one-sided Mann–Whitney–Wilcoxon test compared the level of gene sharing between viruses from two families that share similar hosts and that between viruses from the same two families that have dissimilar hosts.

We further examined the orthogroups shared between viruses of a family pair that infect one major host type (target) but not shared between those that infect the other (reference host type). Each orthogroup was annotated using the NCVOG database as in Section 2.3, the EggNOG v5.0 database (Huerta-Cepas, Szklarczyk, and Forslund et al., 2016) with an auto-adjusted taxonomic scope, or the original published annotations of its member sequences.

## 3. Results

### 3.1 Core-gene phylogeny as a framework of viral lineages and families

**Figure 1.**
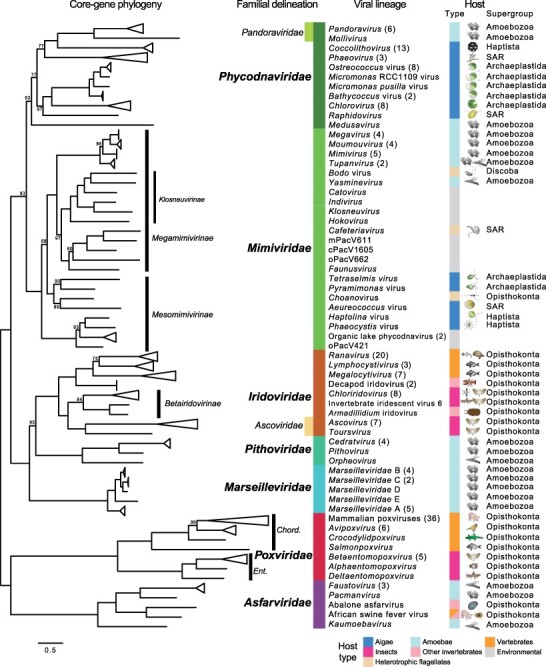
Maximum likelihood phylogeny of 207 NCLDV genomes based on the concatenated alignment of genes encoding five widely distributed proteins: family B DNA polymerase, D5-like helicase-primase, superfamily II helicase, VLTF3-like protein, and DNA-packaging ATPase (Supplementary Table S2). Closely related viruses (numbers indicated in parentheses) that infect similar hosts are collapsed into intrafamilial lineages if possible. Bootstrap nodal support values are only shown for those lower than 100. Viral lineages can be divided into seven major families, which include two smaller families, *Ascoviridae* and *Pandoraviridae*, nested within *Iridoviridae* and *Phycodnaviridae*, respectively. Diagrams of representative hosts, host types, and eukaryotic supergroups of hosts are indicated for each viral lineage. The scale bar shows amino acid sequence divergence in substitutions per site. See Supplementary Table S1 for the full list of virus genomes and Supplementary Figure S1 for the uncollapsed core-gene tree. Chord.: *Chordopoxvirinae*; Ent.: *Entomopoxvirinae.*

A total of 85,833 protein sequences (Supplementary Dataset S1) from 207 complete and near-complete NCLDV genomes (Supplementary Table S1) were included in the OrthoMCL clustering, resulting in 70,878 sequences clustered into 8,710 orthogroups with at least two sequences (Supplementary Dataset S2) and 14,955 unclustered, singleton sequences. We identified orthogroups corresponding to NCVOGs of five widely distributed core proteins (Schulz, Yutin, and Ivanova et al., 2017): family B DNA polymerase, D5-like helicase-primase, superfamily II helicase, VLTF3-like protein, and DNA-packaging ATPase. The sequences in these orthogroups were extracted, aligned, and concatenated into one alignment (Supplementary Dataset S3), from which a maximum likelihood phylogeny of 207 viruses was inferred (Supplementary Fig. S1; Supplementary Dataset S4). To better portray the core-gene-based diversity by avoiding biases in sampling and sequencing, highly related viruses, often those infecting the same hosts, were collapsed into viral lineages that are generally recognized (Fig. 1).

Based on the phylogenetic tree, viral lineages are grouped into seven major clades at the family level: *Asfarviridae, Poxviridae, Marseilleviridae, Pithoviridae, Iridoviridae, Mimiviridae*, and *Phycodnaviridae* (Fig. 1). To maintain the monophyly of each of these family clades, some previously proposed families are placed under larger families in this study unless otherwise noted. *Ascoviridae*, despite its standing in the taxonomy of the International Committee on Taxonomy of Viruses (ICTV) taxonomy (https://talk.ictvonline.org/taxonomy), is classified under *Iridoviridae* for being nested within the latter. Similarly, *Pandoraviridae* (Legendre, Fabre, and Poirot et al., 2018), here including the closely associated *Molliviridae* (Christo-Foroux, Alempic, and Lartigue et al., 2020), is placed within *Phycodnaviridae*. These phylogenetic positions are overall consistent with other core-gene phylogenies (Koonin and Yutin 2018; Guglielmini, Woo, and Krupovic et al., 2019; Needham, Poirier, and Hehenberger et al., 2019). In addition, *Medusavirus*, which formed the proposed monotypic *Medusaviridae* (Yoshikawa, Blanc-Mathieu, and Song et al., 2019), is included here within a well-supported monophyletic *Phycodnaviridae*.

The core-gene phylogeny also provides a framework of intrafamilial relationships. *Poxviridae* is divided into two well-recognized subfamilies, namely the insect-infecting *Entomopoxvirinae* and the vertebrate-infecting *Chordopoxvirinae*. In *Iridoviridae*, the decapod iridoviruses, grouped under the invertebrate subfamily *Betairidovirinae* in the ICTV system, was resolved as sister to the vertebrate subfamily *Alphairidovirinae* (*Ranavirus, Lymphocystivirus*, and *Megalocytivirus*). With the inclusion of some environmental metagenomically assembled genomes (Yau, Lauro, and DeMaere et al., 2011; Schulz, Yutin, and Ivanova et al., 2017; Schulz, Alteio, and Goudeau et al., 2018; Needham, Poirier, and Hehenberger et al., 2019), *Mimiviridae* is comprised by a strongly supported *Megamimivirinae* and a paraphyletic *Mesomimivirinae*, both of which are recently proposed subfamilies (Gallot-Lavallée, Blanc, and Claverie 2017; Mihara, Koyano, and Hingamp et al., 2018). Another proposed subfamily, *Klosneuvirinae* (Schulz, Yutin, and Ivanova et al., 2017), forms a clade nested within *Megamimivirinae*, which is consistent with a previous phylogenetic analysis (Deeg, Chow, and Suttle 2018). Whereas *Mesomimivirinae* contains viruses of haptophyte (Haptista) and chlorophyte (Archaeplastida) algae, viruses with larger genomes that infect amoebae are only found in *Megamimivirinae* (Fig. 1).

At the interfamilial level, the deepest split separates NCLDVs into *Asfarviridae*–*Poxviridae* and the rest of the families (Fig. 1), corresponding to the ICTV classes *Pokkesviricetes* and *Megaviricetes* (Koonin, Dolja, and Krupovic et al., 2020), respectively. The latter is further divided into the MPI (*Marseilleviridae*, (*Pithoviridae* and *Iridoviridae*)) clade and the MP (*Mimiviridae* and *Phycodnaviridae*) clade (Fig. 1). It should be noted that in some studies the sister group of *Iridoviridae* was *Marseilleviridae* instead of *Pithoviridae* (Koonin and Yutin 2018; Guglielmini, Woo, and Krupovic et al., 2019; Moniruzzaman, Martinez-Gutierrez, and Weinheimer et al., 2020a). Previous core-gene analyses encompassing a large number of metagenomically assembled genomes resulted in an MP clade with either both *Mimiviridae* and *Phycodnaviridae* being monophyletic (Schulz, Roux, and Paez-Espino et al., 2020) or a paraphyletic *Phycodnaviridae* where *Mimiviridae* is nested (Moniruzzaman, Martinez-Gutierrez, and Weinheimer et al., 2020a). The phylogenetic tree inferred in this study resolves *Mimiviridae* and *Phycodnaviridae* as two well-separated families, with a bootstrap support of 93 grouping them as the MP clade. Overall, Fig. 1 provides a core-gene-based framework of NCLDV lineages and families, which forms the reference for comparison in gene content analyses.

### 3.2 Clusters of NCLDV genomes based on gene content sharing

**Figure 2.**
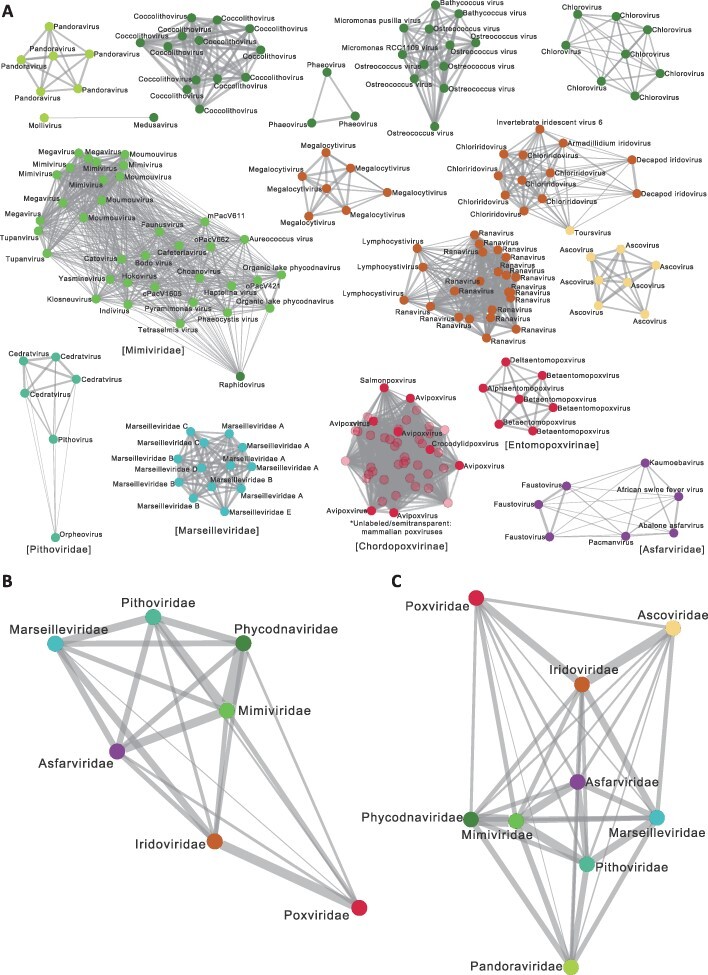
Networks of gene sharing among NCLDV genomes. (A) MCL clusters of individual genomes (nodes) are shown in networks with edges representing gene sharing between genomes. Labels correspond to viral lineages defined in Fig. 1. Supplementary Figure S2 shows IDs of individual genomes (Supplementary Table S1). (B and C) Networks of family-level pangenomes in the seven- (B) or nine-family (C) classification systems. Node colors correspond to families defined in Fig. 1, with *Ascoviridae* and *Pandoraviridae* distinguished in A and C. In each panel, the edge thickness is proportional to the level of gene sharing.

Using MCL clustering based on the level of orthogroup sharing between genomes, the 207 NCLDVs were grouped into 16 clusters, with the relationships in each cluster visualized as a network in Prefuse Force Directed Layout (Fig. 2A and Supplementary Figure S2). Each of the families *Asfarviridae, Marseilleviridae*, and *Pithoviridae* forms a distinct cluster comprised by all and only members of the same family. It suggests that orthogroup sharing between genomes is relatively strong and homogeneous within each of these families, be it overall at high levels as in *Marseilleviridae* or lower levels as in *Asfarviridae* and *Pithoviridae*. Despite having the most diverse eukaryotic hosts and 11 environmental MAG sequences *Mimiviridae* almost forms its own large cluster, with *Raphidovirus* from *Phycodnaviridae* intriguingly co-clustered and loosely connected to the *Mimiviridae* viruses. There is no visible separation between the subfamilies or subclades of *Mimiviridae*, except for stronger connections among genomes of *Megavirus, Moumouvirus, Mimivirus*, and *Tupanvirus*, which are closely related lineages in a strongly supported clade (Fig. 1).

In contrast to families corresponding to single clusters, the other families show higher heterogeneity in gene contents across subfamilies or lineages. *Poxviridae* was grouped into clusters formed by its two subfamilies, *Entomopoxvirinae* and *Chordopoxvirinae*. *Iridoviridae* forms four clusters: *Megalocytivirus*, other viruses in *Alphairidovirinae* (i.e. *Ranavirus* and *Lymphocystivirus*), *Ascovirus*, and all the other invertebrate-infecting viruses (Fig. 1A and Supplementary Fig. S2). It is notable that within *Alphairidovirinae, Megalocytivirus* genomes form their own cohesive group, while *Lymphocystivirus*, also fish viruses, are clustered with the fish- and tetrapod-infecting ranaviruses. What is also interesting is that despite being the sister of *Alphairidovirinae* in the core-gene tree, the decapod iridoviruses were clustered with *Betairidovirinae* and *Toursvirus*, which mainly infect insects.

*Phycodnaviridae* has the highest number of clusters, which roughly correspond to the lineages defined in Fig. 1, including *Pandoravirus, Coccolithovirus, Phaeovirus, Chlorovirus*, and prasinoviruses (viruses of *Bathycoccus, Micromonas*, and *Ostreococcus*). *Mollivirus*, sister to *Pandoravirus* in the core-gene phylogeny (Fig. 1), is clustered with *Medusavirus* at a low level of gene sharing in our MCL analysis (Fig. 2A), whereas these three genera together formed a single clade in a gene content tree (Yoshikawa, Blanc-Mathieu, and Song et al., 2019). The clustering results indicate high gene content heterogeneity across *Phycodnaviridae* lineages, with each of them marked by a distinct gene repertoire that was shaped by unique gain and loss events. Compared with *Phycodnaviridae*, lineages in *Mimiviridae*, which basically form a single large cluster (Fig. 2A), do not have such distinct gene contents, but instead have generally low levels of gene sharing across all lineages and viruses.

It is worth mentioning that the clusters of *Phycodnaviridae* have rather different levels of gene sharing within themselves, which to a large extent reflects the genomic variation in each cluster. For example, among chloroviruses, the difference in genome size or coding capacity can be up to ∼25 per cent (Van Etten, Agarkova, and Dunigan 2020). On the contrary, the genome size variation among the coccolithoviruses is only up to12 per cent (Supplementary Table S1) and their gene contents are largely conserved (Ku, Sheyn, and Sebé-Pedrós et al., 2020). These differences are clearly reflected in the thickness of edges within these two clusters (Fig. 2A) and might be attributed to different sampling efforts for these two lineages or a possible earlier origin of *Chlorovirus* than *Coccolithovirus*. Clustering and network analyses based on gene sharing are therefore useful tools for visualizing highly variable gene contents of NCLDV genomes, showing both lower gene sharing between members of different clusters than the same cluster and variation in within-cluster gene sharing. The clusters in Fig. 2A also clearly demonstrate that gene content variation and heterogeneity in gene sharing patterns of NCLDVs and lineages cannot be directly inferred from the core-gene phylogeny.

### 3.3 Gene-sharing patterns contradict core-gene phylogeny of families

Network analyses can be further applied to study gene sharing patterns among NCLDV families. All viral genomes of each family were treated as one pangenome, encompassing the entire repertoire of orthogroups in that family. Networks were constructed based on the levels of pairwise orthogroup sharing between families, either under the seven-family system as used in this study (Fig. 2B) or with *Ascoviridae* and *Pandoraviridae* as standalone families (Fig. 2C). Here we can clearly see even starker contrasts between the gene-sharing networks and the core-gene phylogeny at the interfamilial level. For example, the core-gene-defined sister families *Poxviridae* and *Asfarviridae*, which form the class *Pokkesviricetes* in the ICTV taxonomy (Koonin, Dolja, and Krupovic et al., 2020), show lower orthogroup sharing between themselves than each of them with some other families (Fig. 2B). In particular, *Poxviridae* has the strongest link to *Iridoviridae*, which in turn has unexpectedly the lowest level of orthogroup sharing with its sister group in the core-gene tree *Pithoviridae*. Families that mainly infect microbial eukaryotes—*Asfarviridae, Pithoviridae, Marseilleviridae, Phycodnaviridae*, and *Mimiviridae*—apparently form a subgroup within the network, showing strong connections among themselves, with the *Phycodnaviridae*–*Mimiviridae* link as the thickest edge in the whole network (Fig. 2B).

The overall pattern is not much different when *Ascoviridae* and *Pandoraviridae* are treated as separate families (Fig. 2C). The strong connection between *Iridoviridae* (excluding *Ascoviridae* members) and *Ascoviridae* is consistent with the nested position of *Ascoviridae* in Iridoviridae in the tree (Fig. 1) and the co-clustering of *Toursvirus* with invertebrate-infecting iridoviruses (Fig. 2A). Despite the nested position of *Pandoraviridae* within *Phycodnaviridae* in the tree (Fig. 1), which suggests they are derived phycodnaviruses (Yutin and Koonin 2013), *Pandoraviridae* does not show much higher gene sharing with *Phycodnaviridae* (excluding *Pandoraviridae* members) but rather have similar connections to *Pithoviridae, Marseilleviridae*, and *Mimiviridae* as well (Fig. 2C). This echoes its unique gene repertoires as shown by the separate clustering of individual pandoravirus genomes (Fig. 2A).

### 3.4 Gene content variation correlates with supergroup-level host diversity

The incompatible patterns between core-gene phylogeny and gene-sharing networks, especially at the interfamilial level, prompted us to investigate the potential effects of host associations on gene content variation and evolution across NCLDV families. The known hosts of NCLDVs are distributed across eukaryotic supergroups (Sun, Yang, and Kao et al., 2020; Meng, Endo, and Blanc-Mathieu et al., 2021)—major lineages and highest taxonomic levels of eukaryotes that are highly divergent in their shared sequences and overall gene contents (Ku, Nelson-Sathi, and Roettger et al., 2015; Adl, Bass, and Lane et al., 2019; Keeling and Burki 2019). Given the large genomic and biological differences across eukaryotic supergroups, we speculated that NCLDV families with more diverse hosts would tend to have higher genomic variation across intrafamilial lineages.

**Figure 3.**
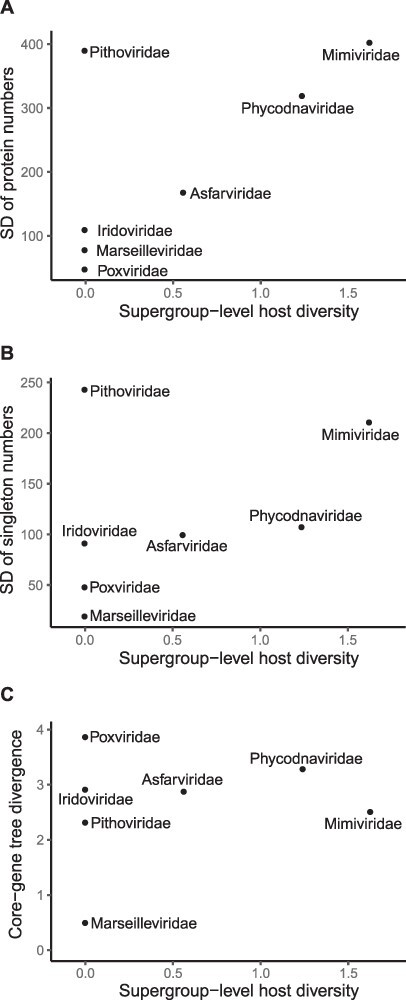
Genomic variation and host diversity of NCLDV families. The supergroup-level host diversity of individual NCLDV families is plotted with measures of genomic variation across lineages in each family, including SD of protein-coding sequence counts (A), SD of unclustered singleton sequence counts (B), and phylogenetic distance (substitutions per site) in the core-gene tree (Fig. 1) (C).

With most of the NCLDVs included in this study having known hosts (Fig. 1), we quantified the supergroup-level host diversity of each family using a Shannon-index-based indicator and calculated three measures of intrafamilial genomic variation (Fig. 3). The standard deviation (SD) of predicted protein-encoding sequences largely correlates with the host diversity index (Fig. 3A). The main exception to this correlation is amoeba-infecting *Pithoviridae*, where the largest genome in *Orpheovirus* (Andreani, Khalil, and Baptiste et al., 2018) has more than 2.5 times the number of protein sequences predicted in the *Pithovirus* genome. Since these two genera represent two of the only three lineages in *Pithoviridae* (Rodrigues, Andreani, and Andrade et al., 2018), gene content variation in this small family is strongly biased by the presence of one large genome.

Compared with total protein counts, less correlation is seen between host supergroup diversity and the SD of singleton numbers (Fig. 3B), which are unclustered sequences and possibly represent unique genes that originate through processes like *de novo* gene creations (Carvunis, Rolland, and Wapinski et al., 2012; Legendre, Fabre, and Poirot et al., 2018). However, this measure could also be biased by differences in gene prediction criteria across studies. Almost no correlation is observed between the core-gene sequence divergence and host diversity of NCLDV families (Fig. 3C). For one thing, *Poxviridae* lineages, which all infect animals (Opisthokonta), have among them the highest pairwise sequence divergence (Fig. 3C). For another, the most host-diverse family *Mimiviridae* tends to have shorter distances between its tips and last common ancestor in both Fig. 1 and previously published core-gene trees (Koonin and Yutin 2018; Guglielmini, Woo, and Krupovic et al., 2019; Schulz, Roux, and Paez-Espino et al., 2020; Moniruzzaman, Martinez-Gutierrez, and Weinheimer et al., 2020a). Overall, it is intrafamilial gene content variation, but not sequence divergence, that correlates with supergroup-level host diversity.

### 3.5 Higher gene sharing among viruses infecting similar host types

**Figure 4.**
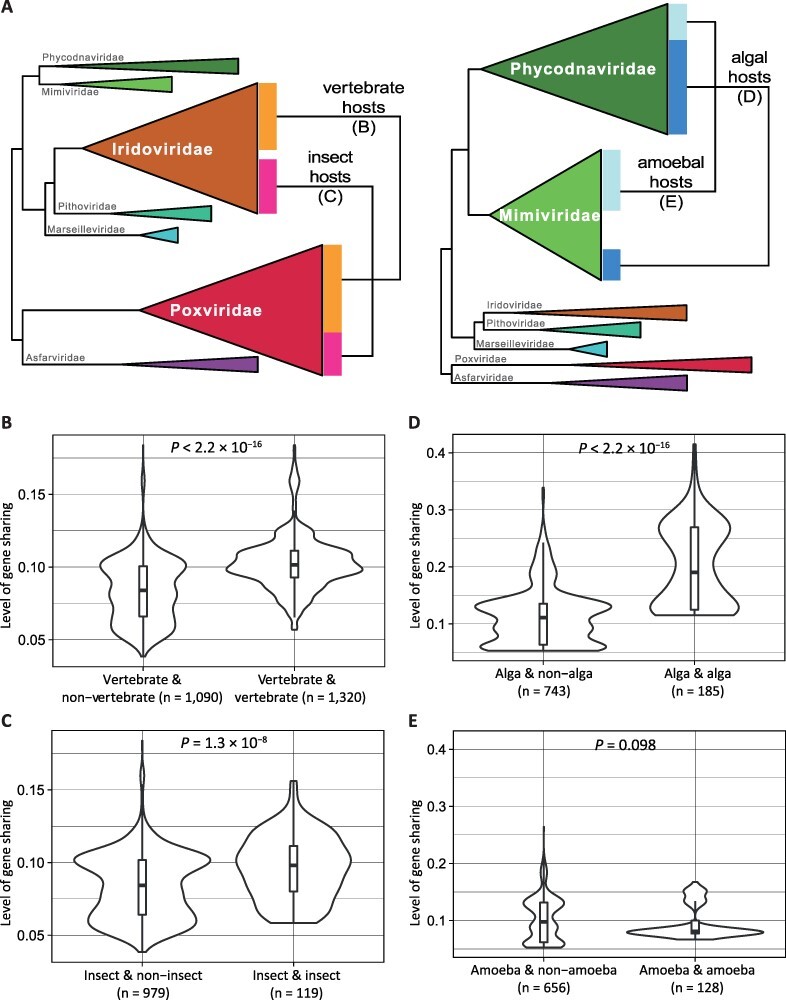
Comparisons of gene sharing among NCLDVs with similar and dissimilar host types. (A) Schematic of our pairwise comparative approach to test the relationships between host associations and gene content sharing. Highlighted family pairs are used for the analyses in B and C (left) and D and E (right), respectively. For each family, colored bars correspond to the proportion of viruses with a specific host type. (B–E) The violin and box plots display the level of gene sharing in all possible pairs of viruses that belong to two families and that infect similar or dissimilar host types. (B and C) *Iridoviridae* and *Poxviridae* viruses that do or do not infect vertebrates (B) or insects (C). (D and E) *Mimiviridae* and *Phycodnaviridae* viruses that do or do not infect algae (D) or amoebae (E). The *P* value of the Mann–Whitney–Wilcoxon test is shown for each comparison, with the number of virus pairs (*n*) indicated in parentheses.

We further employed a comparative approach to investigate the relationships between gene repertoires and host associations. To exclude the effects of viral phylogenetic relatedness on gene sharing, we conducted pairwise comparisons of viral genomes for each of the two pairs of families—*Poxviridae* vs. *Iridoviridae* and *Mimiviridae* vs. *Phycodnaviridae* (Fig. 4A). These two pairs were chosen for having two of the highest levels of interfamilial gene sharing (edge thickness in Fig. 2B). In each pair, there are also a sizable number of viruses with similar and dissimilar hosts in both families, so that it was possible to test whether viruses from the same two families (i.e. viruses with roughly same phylogenetic distance) tend to share more genes when infecting similar hosts. Here instead of supergroups, which are taxa too coarse for the purpose of the analysis, we adopted four host types defined by phylogenetic groupings (vertebrates, insects, and amoebae (Amoebozoa)) or by both phylogenetic and eco-physiological similarities (algae (photosynthetic eukaryotes from Archaeplastida, Haptista, and SAR)).

*Iridoviridae* viruses of vertebrate hosts show significantly higher levels of gene sharing with *Poxviridae* viruses of vertebrate hosts than between vertebrate viruses from one family and nonvertebrate members (all invertebrates) from the other (Fig. 4B). Similarly, pairs of insect viruses from the two families share more genes than pairs of insect and noninsect viruses (Fig. 4C). The difference is more significant in the comparison between algal virus pairs from *Mimiviridae* and *Phycodnaviridae* and algal–nonalgal pairs from the same two families (Fig. 4D). However, higher gene sharing is not found between amoebal viruses of *Mimiviridae* and *Phycodnaviridae* than amoebal–nonamoebal pairs (Fig. 4E). In addition to the lower numbers of virus pairs for amoebal–nonamoebal comparisons, it should be noted that here the ‘amoebal viruses’ are viruses that can infect and be propagated in *Acanthamoeba* or *Vermamoeba*, but most of them have not been directly observed within these amoebae in nature. In other words, the amoebae are lab hosts but not necessarily the natural and the only hosts of these NCLDVs (Francis, Ominami, and Bou Khalil et al., 2019; Sun, Yang, and Kao et al., 2020).

For viruses with known natural hosts (vertebrates, insects, or algae), host similarity is associated with significantly higher proportions of shared orthogroups (Fig. 4). Two possible explanations for this observation are that similar hosts can potentially select for similar genes in their viruses and that similar host genomes or host-associated microbial genomes provide similar pools of genes that can be transferred to viruses. It should be pointed out that the level of orthogroup sharing between viruses of two families with similar host types is generally below 0.3 (i.e. 30 per cent of shared orthogroups) (Fig. 4), suggesting the majority of genes are still unique to individual viral lineages. It is consistent with gene-sharing-based clustering of NCLDV genomes (Fig. 2A), where there is no co-clustering of viruses with similar host types if they represent divergent lineages in the core-gene tree. To summarize, we see correlation between host associations and gene contents but that accounts for only a small proportion of whole gene repertoires, which are mainly genes uniquely acquired during the evolutionary history of individual viral lineages.

### 3.6 Host-related gene families and their predicted functions

**Figure 5.**
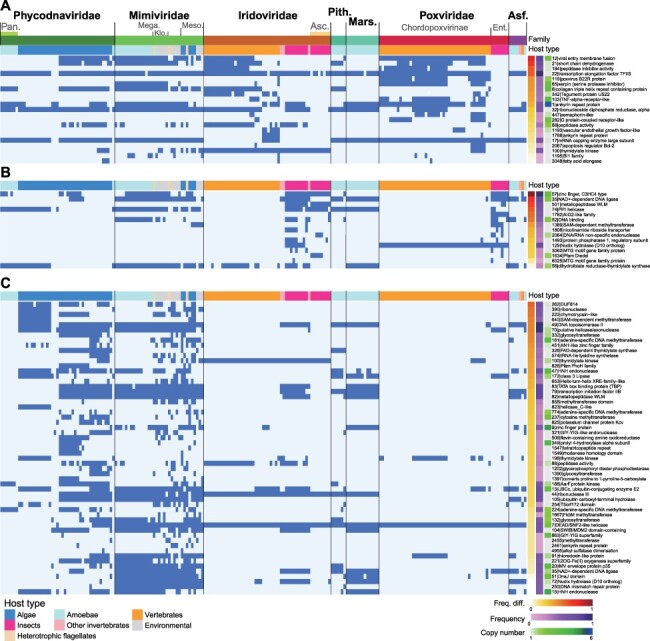
Distribution of orthogroups shared between NCLDVs from different families that infect one of the three target host types: vertebrates, insects, and algae. The presence–absence patterns of these orthogroups (rows; orthogroup|annotation) are shown for 207 NCLDV genomes (columns) in their order in the core-gene tree (Supplementary Figure S1). (A and B) Orthogroups shared between *Iridoviridae* and *Poxviridae* viruses that infect vertebrates (target host type) but not between those infecting insects (reference host type) (A) or vice versa (B). (C) Orthogroups shared between *Mimiviridae* and *Phycodnaviridae* viruses that infect algae but not between those infecting amoebae. Frequency: the proportion of viruses (infecting the target host type) that have a particular orthogroup, averaged across two families. Copy number (color scale in log_2_): average gene copy number in the viruses (infecting the target host type) that have a particular orthogroup, averaged across two families. Freq. diff.: the difference in frequency between viruses infecting target and reference host types. Orthogroups in each panel are sorted by freq. diff., and only those with positive freq. diff. and functional annotations are plotted. For full lists of these orthogroups, see Supplementary Tables S3 and S4. Asc.: *Ascoviridae*; Asf.: *Asfarviridae*; Ent.: *Entomopoxvirinae*; Klo.: *Klosneuvirinae*; Mars.: *Marseilleviridae*; Mega. *Megamimivirinae*; Meso.: *Mesomimivirinae*; Pan.: *Pandoraviridae*; Pith.: *Pithoviridae.*

Our comparative approach also allows for the identification of common orthogroups and gene functions that are associated with specific host types. For vertebrate, insect, and algal viruses in the previous comparisons (Fig. 4), we identified orthogroups uniquely shared by viruses of a specific host type (target) by excluding those also shared by another (reference) host type (Fig. 5).

Some generalized differences in functions can be observed between orthogroups shared by different host types. Genes associated with vertebrate viruses have been noted for their potential roles in apoptosis and immune responses (Iyer, Balaji, and Koonin et al., 2006). These include protein families BI1 (orthogroup 1195) and Bcl-2 (2087) (Fig. 5A), which have antiapoptotic effects (Reimers, Choi, and Bucan et al., 2008), semaphorin (447), which could be involved in immune cell interactions (Takamatsu, Okuno, and Kumanogoh 2010), serpin (serine protease inhibitors) (65) and B22R (119), known to inhibit caspase and apoptosis during poxvirus infection (Brooks, Ali, and Turner et al., 1995; Nichols, De Martini, and Cottrell 2017), and tumor necrosis factor (TNF) alpha receptor (103), which inhibits TNF and block apoptosis (Sedger, Osvath, and Xu et al., 2006; Nichols, De Martini, and Cottrell 2017). These orthogroups are more widely distributed in *Chordopoxvirinae* and mainly found in fish viruses of *Alphairidovirinae* (Fig. 5A). An ankyrin repeat protein family (1) that is the largest orthogroup by sequence count (Supplementary Dataset S2) has the highest copy number per genome averaged across vertebrate poxviruses and iridoviruses (6.12) and is present in variable copy numbers in *Chordopoxvirinae* (9.90), *Megalocytivirus* (2.33), and *Chloriridovirus* (1.00). Ankyrin repeat proteins are involved in various protein interactions, and their role in ubiquitination pathways and suppression of nuclear factor kappa B–mediated antiviral response has been demonstrated in poxviruses (Sonnberg, Seet, and Pawson et al., 2008; Herbert, Squire, and Mercer 2015). In addition, homologs of vascular endothelial growth factor (1193), shown to stimulate blood vessel proliferation underlying the site of infection (Savory, Stacker, and Fleming et al., 2000), are found in a mammalian subclade of *Chordopoxvirinae* (Parapoxvirus, including bovine papular stomatitis virus and orf virus) and fish-infecting *Megalocytivirus* in *Alphairidovirinae*.

Orthogroups shared by insect viruses are mostly related to metabolic activities (Fig. 5B and Supplementary Table S3), including nucleotide metabolism (dihydrofolate reductase-thymidylate synthase) (56), Nudix hydrolase (129), phosphatase (1493), methyltransferase (1389), and AIG2-like family (putative gamma-glutamylcyclotransferase) (1782). The Pif1 helicase (74) in the shared orthogroup list could function in the maintenance and replication of double-stranded DNA (Byrd and Raney 2017). Insect-infecting *Ascovirus* and *Mythimna separata* entomopoxvirus L encode Diedel (1634), which is also endogenously encoded in *Drosophila* and can regulate the antiviral immune deficiency pathway to promote insect survival and likely the success of viral replication (Lamiable, Kellenberger, and Kemp et al., 2016).

Orthogroups shared by *Mimiviridae* and *Phycodnaviridae* algal viruses but not their amoeba-infecting counterparts outnumber those by vertebrate or insect NCLDVs (Fig. 5C and Supplementary Table S4), which is partially due to their larger genome size. A previously reported protein family is potassium ion channel Kcv (825) (Plugge et al., 2000), which has divergent homologs in several algal NCLDV lineages (Kukovetz, Hertel, and Schvarcz et al., 2020). PhoH (phosphate starvation-inducible protein) (826) is part of bacterial Phosphate (Pho) regulon, present in all prasinoviruses as previously reported (Monier, Welsh, and Gentemann et al., 2012), and in this study also detected in *Aureococcus* and *Pyramimonas* viruses in *Mesomimivirinae*. In addition to these marine algal viruses, it is interesting to note that PhoH is commonly encoded by marine phage genomes (Goldsmith, Crosti, and Dwivedi et al., 2011). In the list of orthogroups are also many putative enzymes that merit further investigation, including methyltransferase (640, 181, 858, 774, 237, 224, 1667, and 2455), glycosyltransferase (332, 1390, and 132), rhodanese (thiosulfate sulfurtransferase) (1549), thymidylate kinase (100 and 198), nuclease (390, 47, 321, 44, and 15), and helicase (7 and 823). HNH endonuclease orthogroups (48 and 14) have particularly high copy number per genome (2.42–3.61), possibly due to their homing activity (Stoddard 2011).

Although the level of gene sharing is not significantly higher between *Mimiviridae* and *Phycodnaviridae* amoeba-infecting viruses than between amoebal and non-amoebal viruses (Fig. 4E), there are still 68 genes that are shared by these amoebal viruses from the two families but not by their algal counterparts (Supplementary Table S4). We note several of these shared orthogroups are part of the translation machinery, including translation initiation factors 4E (127) and SUI1 (292) and two orthogroups annotated as tyrosyl-tRNA synthetase (379 and 1402). Only one orthogroup, tRNA-Ile-lysidine synthetase (574), out of the 98 specifically shared by algal viruses is related to translation. This is in agreement with the generally much larger complement of translation system proteins in amoeba-infecting NCLDVs (Koonin and Yutin 2018). Additionally, shared orthogroups in the ubiquitination system imply its importance during viral infection of protists: ubiquitin-conjugating enzyme E2 (13) and ubiquitin carboxyl-terminal hydrolase (105) in algal viruses (also in *Megamimivirinae*) (Fig. 5C) and ubiquitin-activating enzyme E1 (777) in the amoebal shared list (Supplementary Table S4). Among lineages within *Mimiviridae*, it is interesting to note in Fig. 5C that *Klosneuvirinae*, other environmental MAGs, and *Cafeteriavirus* from *Megamimivirinae* tend to share more orthogroups with alga-infecting and other members of *Mesomimivirinae*. This agrees with their spatial distribution pattern in the gene-sharing network of individual genomes (Fig. 2A) and apparently contradicts the core-gene-based grouping of *Klosneuvirinae* with *Megavirus, Moumouvirus, Mimivirus*, and *Tupanvirus* within *Megamimivirinae*.

## 4. Discussion

With the largest and most diverse genomes in the virus world, NCLDVs have been an area of general interest in evolutionary biology. To date phylogenetic trees based on widely distributed core genes have been the most commonly used method to elucidate evolutionary relationships among NCLDVs. They provide an easy-to-use framework for grouping viruses and form the basis of family- and higher-level taxonomy. However, there are caveats to keep in mind when using core-gene trees to represent evolution of NCLDVs. First of all, there are only three proteins strictly shared across all NCLDVs (Koonin and Yutin 2018; Guglielmini, Woo, and Krupovic et al., 2019; Claverie 2020). Even with less stringent criteria, only up to 10 genes have been included for such phylogenetic analyses (Needham, Poirier, and Hehenberger et al., 2019), compared with hundreds of genes used to infer eukaryotic deep phylogeny (Burki, Kaplan, and Tikhonenkov et al., 2016) and dozens for Bacteria and Archaea (Hug, Baker, and Anantharaman et al., 2016). There is also little evidence that these genes have always been vertically inherited throughout their history in NCLDV genomes (Claverie 2020), as suggested by the discrepancies between their single-gene trees. With clustering and networks of gene-repertoire sharing, this study further shows that the core-gene backbone phylogeny could be a poor predictor for overall gene content relationships at the family level and above.

Gene presence–absence patterns have been used to infer trees of NCLDV gene contents in previous studies (Yutin, Wolf, and Koonin 2014; Legendre, Fabre, and Poirot et al., 2018; Needham, Poirier, and Hehenberger et al., 2019; Yoshikawa, Blanc-Mathieu, and Song et al., 2019). We argue that compared with gene-content trees, the combination of MCL clustering and network analyses of gene sharing is a more flexible and comprehensive approach. Instead of just lineage bifurcations, networks can potentially reveal all-to-all connections invisible in trees. This approach can also be easily applied to family-level pangenomes to uncover interfamilial and other higher-level relationships. Therefore it would be especially useful for the investigation of NCLDV genomes, which exhibit profound variation in gene contents. Indeed this study shows that there is not only variation in orthogroup repertoires across viruses, but variable granularity in the distribution of orthogroups across families (Fig. 2). Viruses of *Asfarviridae, Marseilleviridae, Mimiviridae*, and *Pithoviridae* each correspond to single clusters, whether loosely or strongly connected within each family. On the other hand, *Poxviridae, Iridoviridae*, and *Phycodnaviridae* were broken down into smaller clusters at the level of subfamilies or genera. Thus, different levels of genomic cohesion exist in the core-gene-delineated familial or intrafamilial taxa and it can only be revealed through network analyses. A curious case is the co-clustering of all *Mimiviridae* viruses, where there is no clear separation of them into the subfamilies or other subgroups in the core gene tree. This family has been found to be the most abundant and taxon-rich NCLDVs in marine and other environments and potentially associated with diverse eukaryotic microbes (Schulz, Roux, and Paez-Espino et al., 2020; Moniruzzaman, Martinez-Gutierrez, and Weinheimer et al., 2020a; Meng, Endo, and Blanc-Mathieu et al., 2021). The more homogeneous gene sharing suggests that a large proportion of the *Mimiviridae* ancestral gene repertoire could have been passed down to its descendant lineages during their evolutionary radiation.

We further showed that interfamilial gene sharing does not follow core-gene branching patterns, which forms the basis of ICTV taxonomy. Families in the same higher-level taxon, such as *Pokkesviricetes* (*Poxviridae* and *Asfarviridae*), might not have stronger gene sharing as their core-gene-based grouping would suggest. These discrepancies can be in part attributed to associations with different eukaryotic hosts. Family-level host diversity better correlates with gene content variation rather than core-gene sequence divergence (Fig. 3), and NCLDVs with similar hosts tend to share more genes depending on the host types, including vertebrates, insects, and algae (Fig. 4), such as genes related to host defense in animal viruses or ion transport in algal viruses (Fig. 5). In particular, stronger gene sharing by algal viruses is consistent with the grouping of NCLDVs with phototrophic hosts within *Phycodnaviridae* and *Mimiviridae*, respectively, in gene-content-based hierarchical clustering (Needham, Poirier, and Hehenberger et al., 2019). Many genes have been suggested to be transferred from eukaryotic hosts or other microbes to NCLDVs (Sun, Yang, and Kao et al., 2020). Our analyses identified those genes that might have been convergently transferred to distantly related viral lineages in similar host or environmental settings. Future research on these shared genes can further shed light on common strategies of NCLDVs in different host types or environments.

Based on the gene sharing networks and comparison of host association in this study, NCLDV gene contents can be roughly divided into three categories: (1) a few core genes involved in key processes of viral replication that are common to the vast majority of NCLDVs; (2) dozens of genes shared across divergent viral lineages with the same type of hosts (Fig. 5); and (3) 100 or more genes accumulated during the evolution of a specific viral lineage with a narrow host range. Category 3 comprises the majority of NCLDV genes, and it contributes to distinct gene repertoires and thus separate clustering of certain intrafamilial lineages in Fig. 2A. Even for viruses with the same host (e.g. *Suipoxvirus*/African swine fever virus), each divergent viral lineage represents a unique way to adapt to the host and thus a largely different set of genes. Although large gene repertoires might suggest many genes are dispensable, most genes in NCLDV genomes actually seem to be under purifying selection (Doutre, Philippe, and Abergel et al., 2014; Legendre, Fabre, and Poirot et al., 2018), indicating they are all likely an integral part of the viral replication cycle.

Gene contents largely determine the biology of giant viruses and thus their ecological roles and important aspects of giant virus–eukaryote evolution (Ku 2021). Here we show a global view of giant virus gene content variation, linking gene repertoires and hosts across NCLDV lineages and taxa. This implies that gene contents can reveal present or maybe past host associations, as has been done through the use of putative lateral gene transfers to infer host associations or to verify host predictions (Endo, Blanc-Mathieu, and Li et al., 2020; Schulz, Roux, and Paez-Espino et al., 2020; Meng, Endo, and Blanc-Mathieu et al., 2021). However, host genomes might not be the only source of lateral transfer for NCLDVs. The relative contributions of hosts and other microbes (e.g. host-associated bacteria) to NCLDV genomes still remain to be uncovered. The circumstances of such transfers are also poorly understood, but insights might be gained through further comparative analyses between viruses associated with different host lifestyles (e.g. phagotrophy and autotrophy), host microbial loads, and ecosystems. Another major outstanding question is how the accrued genes, including *de novo* created ones, became integrated into the genomes in different viral lineages, which would be a key molecular mechanism contributing to their plasticity and gigantism.

In summary, this study dissected gene content variation of NCLDVs, or the virus phylum Nucleocytoviricota, at levels from individual genomes to interfamilial relationships. We provide an updated view of the phylogenetic relationships of NCLDVs based on the widely distributed proteins, which helps place recently sequenced NCLDV lineages into the core-gene-based framework of families and lineages. Networks and comparative analyses based on gene sharing between genomes reveal patterns of genomic variation hidden from the core-gene phylogeny. We also report genes associated with specific host types, which would be a useful resource for future functional analyses and experiments. With the ever-increasing number of NCLDV genomes from various ecosystems and the prospect of eventually identifying their individual hosts, we believe the comprehensive approach in this study will further better our understanding of the interactions and coevolution between NCLDVs and eukaryotes.

## Data availability

The datasets generated in this study are available in Supplementary Data, as detailed in the main text. R codes for performing the analyses are deposited on GitHub (https://github.com/TsuWangSun/VirusEvolution2021).

## Supplementary Material

veab081_Supp
