# Supplementary figures and images for: Unraveling gene content variation across eukaryotic giant viruses based on network analyses and host associations
Source: Virus Evol. 2021 Sep 16;7(2):veab081. doi: 10.1093/ve/veab081 (PMC8570155; doi:10.1093/ve/veab081)

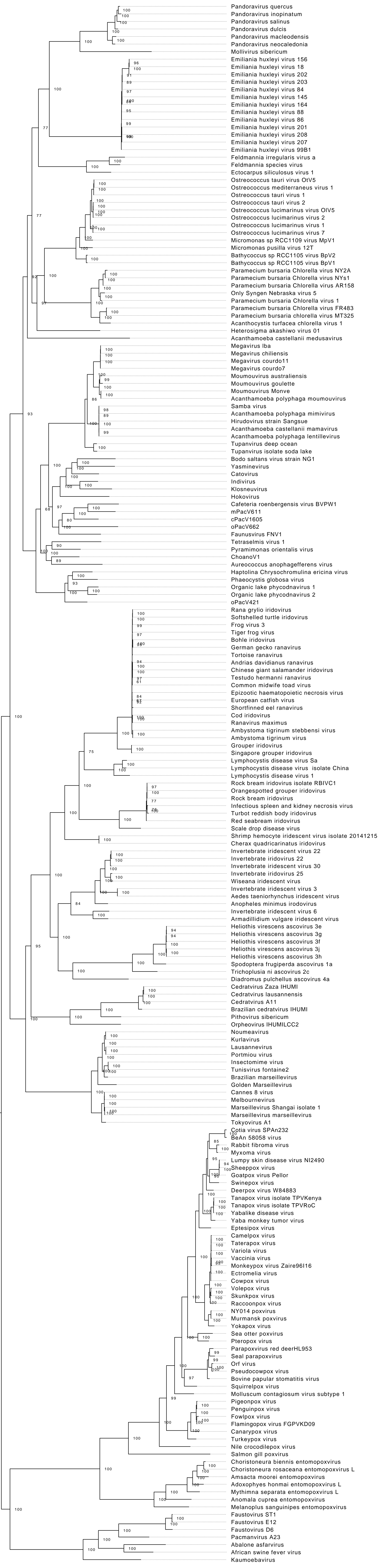

Supplement: veab081_Supp [file veab081_supp.zip › SupplementaryFigureS1.Uncollapsed_phylogeny_v2.pdf]

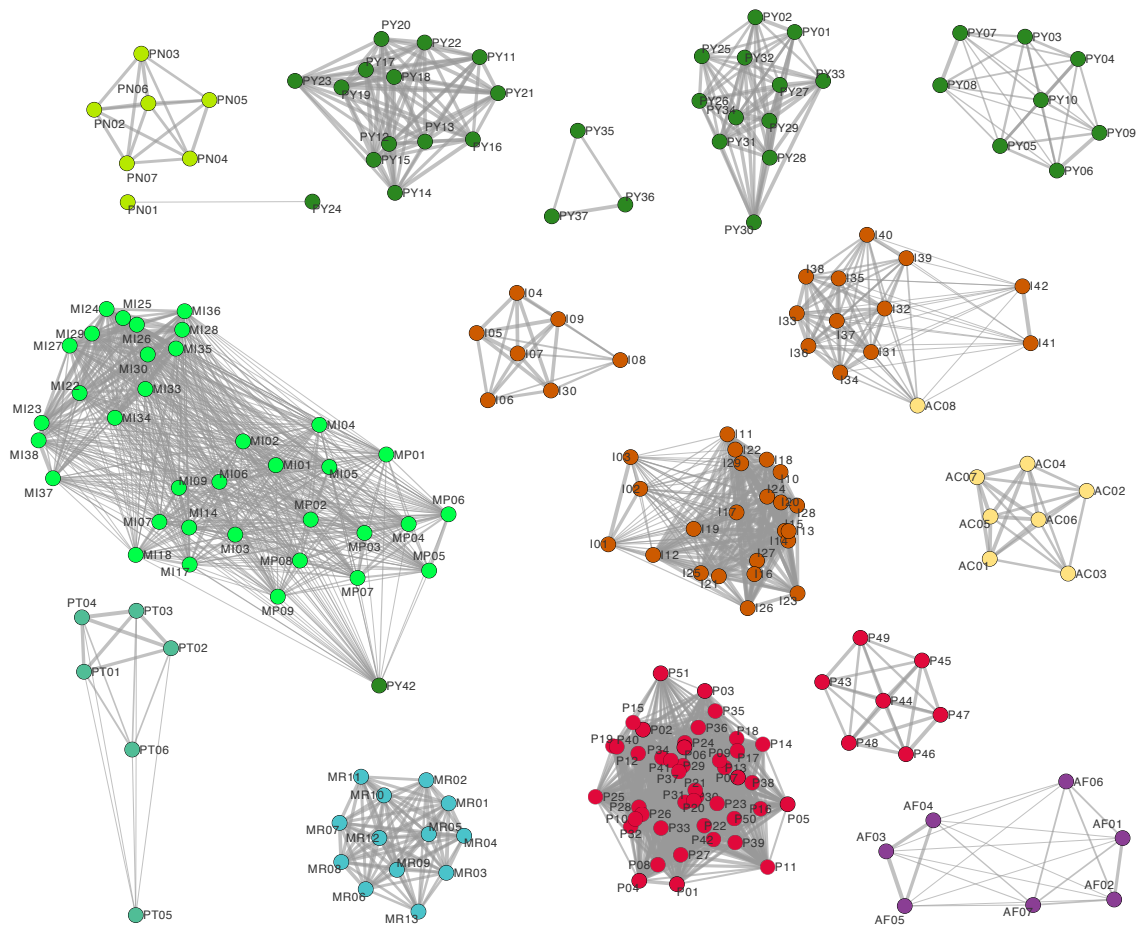

Supplement: veab081_Supp [file veab081_supp.zip › SupplementaryFigureS2.Figure2_strainID.pdf]
